# Maternal Diabetes-Induced Suppression of Oxytocin Receptor Contributes to Social Deficits in Offspring

**DOI:** 10.3389/fnins.2021.634781

**Published:** 2021-02-09

**Authors:** Jianbo Liu, Yujie Liang, Xing Jiang, Jianchang Xu, Yumeng Sun, Zichen Wang, Ling Lin, Yanbin Niu, Shiqi Song, Huawei Zhang, Zhenpeng Xue, Jianping Lu, Paul Yao

**Affiliations:** ^1^Department of Child Psychiatry, Kangning Hospital of Shenzhen, Shenzhen Mental Health Center, Shenzhen, China; ^2^Department of Biomedical Engineering, Southern University of Science and Technology, Shenzhen, China

**Keywords:** autism spectrum disorders, maternal diabetes, oxidative stress, oxytocin receptor, social deficit

## Abstract

Autism spectrum disorders (ASD) are a group of neurodevelopmental disorders characterized by impaired skills in social interaction and communication in addition to restricted and repetitive behaviors. Many different factors may contribute to ASD development; in particular, oxytocin receptor (OXTR) deficiency has been reported to be associated with ASD, although the detailed mechanism has remained largely unknown. Epidemiological study has shown that maternal diabetes is associated with ASD development. In this study, we aim to investigate the potential role of OXTR on maternal diabetes-mediated social deficits in offspring. Our *in vitro* study of human neuron progenitor cells showed that hyperglycemia induces OXTR suppression and that this suppression remains during subsequent normoglycemia. Further investigation showed that OXTR suppression is due to hyperglycemia-induced persistent oxidative stress and epigenetic methylation in addition to the subsequent dissociation of estrogen receptor β (ERβ) from the OXTR promoter. Furthermore, our *in vivo* mouse study showed that maternal diabetes induces OXTR suppression; prenatal OXTR deficiency mimics and potentiates maternal diabetes-mediated anxiety-like behaviors, while there is less of an effect on autism-like behaviors. Additionally, postnatal infusion of OXTR partly, while infusion of ERβ completely, reverses maternal diabetes-induced social deficits. We conclude that OXTR may be an important factor for ASD development and that maternal diabetes-induced suppression of oxytocin receptor contributes to social deficits in offspring.

## Introduction

Autism spectrum disorders (ASD) are a group of neurodevelopmental disorders characterized by deficits in social interaction and communication in addition to restricted and repetitive behaviors (Rossignol and Frye, 2012; Baron-Cohen et al., 2019). Many factors, including genetics/epigenetics, sex and environmental factors have been reported to be associated with ASD development (Rossignol and Frye, 2012; Bralten et al., 2018). We have previously reported that prenatal hormone exposure (Zou et al., 2017; Li et al., 2018; Xie et al., 2018; Xiang et al., 2020) and maternal diabetes (Xiang et al., 2018; Wang et al., 2019) contribute to ASD development, although the detailed mechanism for the etiology of ASD remains largely unknown and various other factors may still need to be investigated.

Oxytocin is a central nervous neuropeptide that is involved in a variety of physiological processes (Marotta et al., 2020) and is mainly synthesized in neurons of the PVN and supraoptic nuclei (SON) in the hypothalamus (Tang et al., 2020). OXTR is widely expressed in human tissues, with particularly high levels being located in limbic brain regions (Kudwa et al., 2014). In conjunction with OXT, OXTR has been reported to regulate diverse social behaviors (Maejima et al., 2018; Gulliver et al., 2019; Resendez et al., 2020; Soltys et al., 2020) and play a role in ASD etiology (Jacob et al., 2007; LoParo and Waldman, 2015; Uzefovsky et al., 2019), although there has been some controversy with these conclusions (Tansey et al., 2010). Epigenetic modification of OXTR has been widely reported to be associated with ASD development (Jack et al., 2012; Maud et al., 2018; Krol et al., 2019; Tops et al., 2019), although the detailed mechanism remains unclear.

Estrogen receptor β (ERβ), together with estrogen receptor α (ERα), is widely expressed in various areas of the brain (Bodo and Rissman, 2006; Phan et al., 2015), and ERβ specifically has been reported to be associated with ASD development and anxiety-related behaviors (Krezel et al., 2001; Crider et al., 2014; Zou et al., 2017). Additionally, ERβ is responsible for the basal expression of superoxide dismutase 2 (SOD2) and estrogen-related receptor α (ERRα) through ERE, subsequently regulating oxidative stress and mitochondria function (Li et al., 2015; Kong et al., 2016). ERβ is colocalized within the PVN and highly expressed in OXT-containing neurons located in hypothalamic regions. Both OXT (Acevedo-Rodriguez et al., 2015) and OXTR (Murata et al., 2014) have been reported to be regulated by ERβ either directly or indirectly; and our recent work showed that maternal diabetes suppresses ERβ expression in brain (Wang et al., 2019), thus, ERβ may play a role in modulating maternal diabetes-mediated social behaviors (Clipperton-Allen et al., 2012; Kudwa et al., 2014).

In this study, we aim to investigate the potential role of OXTR on maternal diabetes-mediated social deficits. Our *in vitro* study in human neuron progenitor cells showed that OXTR expression was suppressed by transient high glucose levels and remained low during subsequent normoglycemia through hyperglycemia-mediated consistent oxidative stress. Further investigation found that OXTR suppression is due to hyperglycemia-mediated epigenetic changes on the OXTR promoter and subsequent dissociation of ERβ from the OXTR promoter. *In vivo* mouse study showed that prenatal OXTR deficiency potentiates maternal diabetes-mediated anxiety-like behavior, while it has little effect on ALB. In addition, postnatal infusion of OXTR reversed maternal diabetes-mediated anxiety-like behavior, while it had little effect on ALB; on the other hand, postnatal infusion of ERβ completely reversed maternal diabetes-mediated social deficits. We conclude that maternal diabetes-induced suppression of oxytocin receptor contributes to social deficits in offspring.

## Materials and Methods

A detailed description can be found in Supplementary Data 1, and the related primers used in this study were shown in Supplementary Table 1.

### Reagents and Materials

Human neural progenitor cells (NPC, #ACS-5003) were obtained from ATCC and were cultured in NPC medium as described previously (Wang et al., 2019). The mouse primary amygdala neurons were isolated and cultured in DMEM medium plus 10% fetal bovine serum (FBS), 10% heat-inactivated defined horse serum, 20 mM D-glucose and 100 U/ml Pen/Strep (from Invitrogen). All cells were maintained in a humidified incubator with 5% CO_2_ at 37°C. In some experiments, the cells were conditionally immortalized using a hTERT lentivirus vector with an extended life span to achieve higher transfection efficiency and experimental stability (Bodnar et al., 1998; Kong et al., 2016).

The antibodies for β-actin (sc-47778), C/EBPα (sc-365318), GATA1 (sc-266), SOD2 (sc-30080), Sp1 (sc-17824) and YY1 (sc-7341) were obtained from Santa Cruz Biotechnology. Antibodies for OXTR (#BS-1314R) was purchased from Fisher; OXT (#AB911) was purchased from Sigma; 8-oxo-dG (4354-MC-050) was purchased from Novus Biologicals; NeuN (#24307) was purchased from Cell Signaling. Antibodies for acetyl-histone H4 K5, K8, K12, and K16 (H4K5,8,12,16ac, #PA5-40084) were obtained from Invitrogen. Antibodies for ERα (ab3575), ERβ (ab3576), anti-histone H3 acetyl K9, K14, K18, K23, K27(H3K9,14,18,23,27ac, ab47915), H4K20me1 (ab9051), H4K20me3 (ab9053), H4R3me1 (ab17339), H3K9me2 (ab1220), H3K9me3 (ab8898), H3K27me2 (ab24684), and H3K27me3 (ab6002) were obtained from Abcam. 3-nitrotyrosine (3-NT) was measured using the 3-Nitrotyrosine ELISA Kit (ab116691 from Abcam) per manufacturers’ instructions. The mitochondrial fraction was isolated using a Pierce Mitochondria Isolation Kit (Pierce Biotechnology) per manufacturers’ instructions. Protein concentration was measured using the Coomassie Protein Assay Kit (Pierce Biotechnology). Luciferase activity assay was carried out using the Dual-Luciferase^TM^ Assay System (Promega) and the transfection efficiency was normalized using a cotransfected renilla plasmid (Zhang et al., 2017). Streptozocin (STZ, #18883-66-4) were obtained from Sigma.

### Construction of OXT/OXTR Reporter Plasmid

Human genomic DNA was prepared from NPC cells. In order to construct OXT/OXTR reporter plasmids, the gene promoter (2 kb upstream of the transcription start site plus first exon) was amplified from Ensembl gene ID: OXT-201 ENST00000217386.2 (for OXT) and OXTR-201 ENST00000316793.7 (for OXTR) by PCR and subcloned into the pGL3-basic vector (# E1751, Promega) using underlined restriction sites with the following primers: OXT forward: 5′-gcgc-acgcgt- ttg gat gcg ggc cac ctg gga -3′ (*Mlu*I) and OXT reverse: 5′- gtac- aagctt- ctt gcg cac gtc gag gtc cgg -3′ (*Hin*dIII); OXTR forward: 5′-gcgc- ggtacc - tgg aac ttt gag gat ttt ttt -3′ (*Kpn*I) and OXTR reverse: 5′- gtac- aagctt - ctg cac cga gtc cgc agg cga -3′ (*Hin*dIII). To map OXTR promoter activity, the related deletion promoter constructs were generated by PCR methods and subcloned into the pGL3-basic vector. All the vectors were verified by sequencing, and detailed information on these plasmids is available upon request (Zhang et al., 2017).

### Generation of Expression Lentivirus

The lentivirus for human ERβ and SOD2 was prepared as described previously in our lab (Wang et al., 2019). The cDNA for mouse ERβ and OXTR was obtained from Open Biosystems and subcloned into the pLVX-Puro vector (from Clontech) using underlined restriction sites with the following primers: mouse ERβ forward primer: 5′- gtac- ctcgag- atg tcc atc tgt gcc tct tct -3′ (Xho1) and mouse ERβ reverse primer: 5′- gtac- tctaga- tca ctg tga ctg gag gtt ctg -3′ (Xba1); mouse OXTR forward primer: 5′- gtac - gaattc- atg gag ggc acg ccc gca gcc -3′ (EcoR1) and mouse OXTR reverse primer: 5′- gtac - tctaga- tca tgc cga gga tgg ttg aga -3′ (Xba1). The lentivirus for ERβ, OXTR, or empty control (CTL) was expressed through Lenti-X^TM^ Lentiviral Expression Systems (from Clontech) per manufacturers’ instructions (Wang et al., 2019).

### Gene Knockdown by shRNA Lentivirus Particles

The shRNA lentivirus particles for human ERβ and SOD2 were prepared as described previously in our lab (Wang et al., 2019). The shRNA lentivirus plasmids for human SOD2 (sc-41655-SH), ERβ (sc-35325-SH) or non-target control (sc-108060) were purchased from Santa Cruz Biotechnology, and the related lentivirus for either ERβ and SOD2 or empty control (CTL) were expressed through Lenti-X^TM^ Lentiviral Expression Systems (from Clontech) per manufacturers’ instructions. The purified and condensed lentivirus were used for *in vitro* gene knockdown. The knockdown efficiency was confirmed by more than 65% of mRNA reduction compared to the control group in cells using real time PCR (see Supplementary Table 1).

### *In vivo* Mouse Experiments

The animal protocol conformed to US NIH guidelines (Guide for the Care and Use of Laboratory Animals, No. 85-23, revised 1996), and was reviewed and approved by the Institutional Animal Care and Use Committee from Kangning Hospital of Shenzhen. All the experimental mice were either OXTR wild type (WT) or OXTR null (OXTR^–/–^) mice with a C57BL/6J mixed genetic background (a kind gift from Dr. Haimou Zhang from Hubei University, China). In the generation of diabetic mice, adult (3-month-old) female mice with either WT or OXTR^–/–^ backgrounds were monitored for estrous cycles with daily vaginal smears. Only mice with at least two regular 4- to 5-day estrous cycles were included in the studies. Chronic diabetic female mice were induced by injection of 35 mg/kg streptozocin (STZ, 0.05 M sodium citrate, pH 5.5) after an 8-h fasting period. Animals with blood glucose >250 mg/dl were considered positive with the success rate of ∼90%, while control (CTL) mice received only vehicle injection (Williams et al., 2017).

#### Mouse Protocol 1 for Prenatal Treatment of Diabetes or OXTR Deficiency

Verified pregnant dams were randomly assigned to the following four groups: Group 1: CTL group mice with OXTR WT background (CTL/WT); Group 2: STZ mice with OXTR WT background (STZ/WT); Group 3: CTL group mice with OXTR null background (CTL/OXTR^–/–^); Group 4: STZ mice with OXTR null background (STZ/OXTR^–/–^). Neurons from the amygdala were isolated on embryonic day 18 (E18) as described below. The male offspring were separated from the dams on day 21 and fed with normal chow until 7–8 weeks old for behavior tests. Then, the offspring were sacrificed and various brain tissues, including the amygdala, hypothalamus and hippocampus, were isolated, flash frozen in dry ice, and then stored in a −80°C freezer for analysis of gene expression and oxidative stress.

#### Mouse Protocol 2 for Postnatal Manipulation of OXTR/ERβ Expression

The male offspring (6 weeks old) from either the CTL or STZ group in Mouse Protocol 1 were anesthetized with a mixture of ketamine (90 mg/kg) and xylazine (2.7 mg/kg) and implanted with a guide cannula targeting the amygdala (26 gauge; Plastics One) (Neal-Perry et al., 2014). The following stereotaxic coordinates from the bregma were used for the amygdala: anteroposterior (AP) = −1.4, mediolateral (ML) = ±3.5, dorsoventral (DV) = −5.1. Dorsoventral coordinates, which were based on the mouse brain atlas (Heldt and Ressler, 2006), were measured from the skull surface with the internal cannula extending 2 mm beyond the end of the guide cannula. The cannula was attached to the skull with dental acrylic and jeweler’s screws and closed with an obturator (Hu et al., 2015). An osmotic minipump (Alzet model 2002; flow rate 0.5 μl/h; Cupertino, CA, United States) connected to a 26-gauge internal cannula that extended 1 mm below the guide was implanted and used to deliver ORTR overexpression (↑OXTR), ERβ overexpression (↑ERβ), or vehicle (VEH) lentivirus. Vehicle consisting of artificial cerebrospinal fluid (aCSF; 140 mM NaCl, 3 mM KCl, 1.2 mM Na2HPO4, 1 mM MgCl2, 0.27 mM NaH2PO4, 1.2 mMCaCl2, and 7.2 mM dextrose, pH 7.4) was used for the infusion of the lentivirus. Infusion (flow rate 0.5 μl/h) begun immediately after placement of the minipump. 0.5 μl of total 2 × 10^3^ cfu of lentivirus was infused for 1 h. The experimental mice were separated into four groups, with 10 in each group. Group 1: CTL offspring with vehicle control lentivirus infusion (CTL/P-VEH); Group 2: STZ offspring with vehicle control lentivirus infusion (STZ/P-VEH); Group 3: STZ offspring with OXTR expression lentivirus infusion (STZ/P-↑OXTR); Group 4: STZ offspring with ERβ expression lentivirus infusion (STZ/P-↑ERβ). Cannula placement was verified histologically postmortem by the injection of 0.5 μl of India ink (volume matching that of drug delivery in the experiments). Mice whose dye injections were not located in the amygdala were excluded from the data analysis. Two weeks after lentivirus infusion, the offspring were used for behavior tests followed by biomedical analysis, as indicated in Mouse Protocol 1 (Zou et al., 2017).

### DNA Methylation Analysis

We developed a real-time PCR-based method for methylation-specific PCR (MSP) analysis on the human OXTR promoter according to the previously described method with some modifications (Eads et al., 2000; Ogino et al., 2006; Nosho et al., 2008). The genomic DNA from human #ACS-5003 cells was extracted and purified before then being treated by bisulfite modification using the EpiJET Bisulfite Conversion Kit (#K1461, Fisher). The modified DNA was then amplified using methylated and unmethylated primers for MSP that were designed using the Methprimer software^1^ with the below details: Methylated primer: forward 5′- ttt gag ttt att gtt aaa gtc gt -3′, reverse 5′- aaa taa taa tat tct tcc ccg aa -3′; Unmethylated primer: forward 5′- ttt gag ttt att gtt aaa gtt gt -3′; reverse 5′- aaa taa taa tat tct tcc cca aa -3′. Product size: 147 bp (methylated) and 147 bp (unmethylated); CpG island size: 134 bp; Tm: 64.2°C. The final methylation readout was normalized by unmethylated input PCR (Zou et al., 2017).

### Animal Behavior Test

The animal behavior test of offspring was carried out at 7–8 weeks of age. Anxiety-like behavior was evaluated using the marbles burying tests (MBT) and the elevated plus maze (EPM) tests (Zou et al., 2017; Xie et al., 2018). ALB was evaluated using ultrasonic vocalization (USV), social interaction (SI) tests and a three-chambered social test as described below (Moy et al., 2004; Silverman et al., 2010; Schaafsma et al., 2017).

### Statistical Analysis

The data was given as mean ± SEM and all the experiments were performed at least in quadruplicate unless indicated otherwise. The one-way analysis of variance (ANOVA) followed by the Turkey–Kramer test was used to determine statistical significance of different groups, and the two-way ANOVA followed by the Bonferroni *post hoc* test was used to determine the differences of two factors (e.g., OXTR deficiency and maternal diabetes) using SPSS 22 software, and a *P* value of <0.05 was considered significant (Li et al., 2019; Zhou et al., 2019).

## Results

### Transient High Glucose Causes Persistent OXTR Suppression During Subsequent Normoglycemia Through Hyperglycemia-Mediated Consistent Oxidative Stress

**FIGURE 1:**
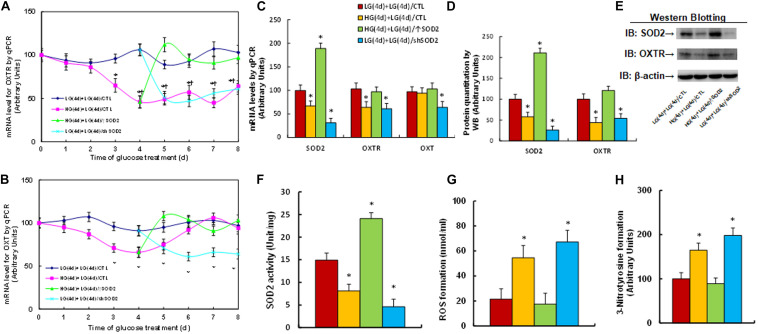
Transient high glucose causes persistent OXTR suppression during subsequent normoglycemia through hyperglycemia-mediated consistent oxidative stress. Human ACS-5003 neurons were treated with either 5 mM low glucose (LG) or 25 mM high glucose (HG) for 4 days. The cells were then infected by empty (CTL), SOD2 overexpression (↑SOD2), or SOD2 knockdown (shSOD2) lentivirus for 1 day before they were then treated by LG for another 4 days in the presence of 1% serum; the cells were then harvested for further analysis. **(A,B)** Cells were harvested at different time points for analysis of mRNA levels. **(A)** OXTR levels; **(B)** OXT levels; *n* = 4, ^∗^*P* < 0.05, vs. day 0 group; ^¶^ *P* < 0.05, vs. day 3 group. **(C–H)** Cell were harvested on day 8 for biomedical analysis. **(C)** mRNA levels, *n* = 4. **(D)** Quantitation of protein levels, *n* = 5. **(E)** Representative western blotting pictures for **(D)**. **(F)** SOD2 activity, *n* = 5. **(G)** ROS formation, *n* = 5. **(H)** 3-nitrotyrosine formation, *n* = 5. ^∗^*P* < 0.05, vs. LG(4d) + LG(4d)/CTL group. Data were expressed as mean ± SEM.

We first evaluated the potential effect of glucose memory on the gene expression of OXTR and OXT. Human ACS-5003 neurons were first treated by high glucose (25 mM HG) for 4 days before remaining in low glucose levels (5 mM LG) for another 4 days. The results showed that 4-day high glucose treatment significantly suppressed the gene expression of both OXTR (see Figure 1A) and OXT (see Figure 1B); when the cells switched into low glucose, OXTR expression remained low, while OXT expression returned to normal; SOD2 expression (↑SOD2) on day 5 completely reversed the HG-mediated effect; and SOD2 knockdown (shSOD2) on day 5 mimicked the HG-mediated effect. Furthermore, the mRNA levels for OXTR and OXT on day 8 were presented in Figure 1C in addition to mRNA levels of SOD2, indicating that SOD2 mRNA expression was suppressed in HG and remained low during subsequent LG. In addition, the manipulation of SOD2 using lentivirus was successful; SOD2 expression lentivirus (↑SOD2) significantly increased, while SOD2 knockdown lentivirus (shSOD2) significantly decreased, SOD2 mRNA levels (see Figure 1C). We also measured the protein levels for SOD2 and OXTR, and an expression pattern similar to that of the mRNA was observed (see Figures 1D,E and Supplementary Figure 1a). On the other hand, we could not detect the presence of OXT proteins by western blotting, indicating that OXT protein is not expressed in ACS-5003 neurons. We then measured the SOD2 activity, and the results showed a pattern similar to that of SOD2 mRNA (see Figure 1F). Finally, we evaluated oxidative stress, and the results showed that ROS formation significantly increased in the HG(4d) + LG(4d)/CTL group (see Figure 1G). 3-nitrotyrosine formation (see Figure 1H) also increased compared to the LG(4d) + LG(4d)/CTL group, and SOD2 expression HG(4d) + LG(4d)/↑SOD2 completely reversed, while SOD2 knockdown LG(4d) + LG(4d)/shSOD2 mimicked, the high glucose-mediated effect. Our results indicate that transient high glucose causes persistent OXTR suppression during subsequent normoglycemia through hyperglycemia-mediated consistent oxidative stress.

### Hyperglycemia Induces OXTR Suppression Through Epigenetic Modification and the Subsequent Dissociation of ERβ From the OXTR Promoter

**FIGURE 2:**
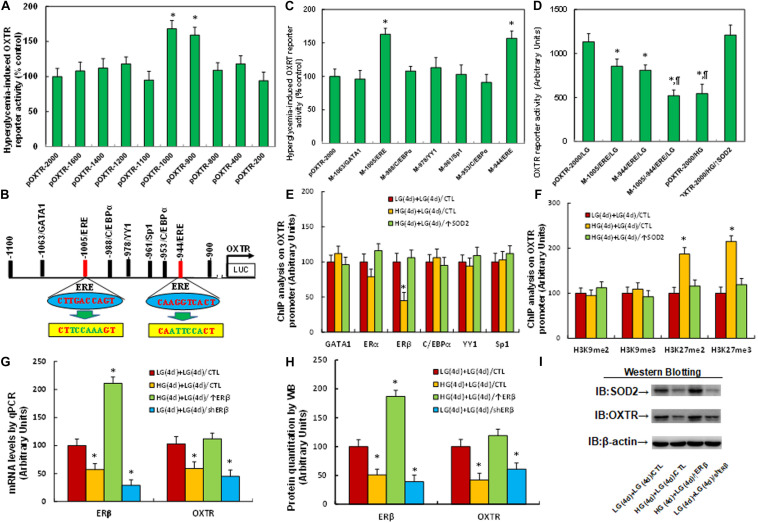
Hyperglycemia induces OXTR suppression through epigenetic modification and the subsequent dissociation of ERβ from the OXTR promoter. **(A)** The conditional immortalized ACS-5003 neurons were transiently transfected with either OXTR full length (pOXTR-2000) or deletion reporter plasmids. After 24 h, the cells were treated with either 5 mM low glucose (LG) or 25 mM high glucose (HG) for 3 days and the OXTR reporter activities were calculated, *n* = 5. ^∗^*P* < 0.05, vs. pOXTR-2000 group. **(B)** The schematic picture for the potential transcriptional binding motif in the range of –900∼1100 (from transcription start site) on the OXTR promoter with two potential ERE binding sites marked in red as well as related mutation sites marked in green. **(C)** The cells were transiently transfected by either a wild type OXTR reporter construct (pOXTR-2000) or single point mutation at the site shown in panel **(B)**, and then treated with either LG or HG for 3 days, and the OXTR reporter activities were calculated, *n* = 5. ^∗^*P* < 0.05, vs. pOXTR-2000 group. **(D)** The cells were transiently transfected by OXTR full length (pOXTR-2000), single mutant, or double mutations as indicated, or infected by SOD2 lentivirus (↑SOD2), and then treated with either LG or HG for 3 days; the OXTR reporter activities were then calculated, *n* = 5. ^∗^*P* < 0.05, vs. pOXTR-2000/LG group; ^¶^ *P* < 0.05, vs. M-1005/ERE/LG group. **(E,F)** Cells were treated by either 4-day LG plus 4-day LG [LG(4d) + LG(4d)], or 4-day HG plus 4-day LG [HG(4d) + LG(4d)], or the cells were infected on day 4 by SOD2 lentivirus [HG(4d) + LG(4d)/↑SOD2]; the cells were then used for ChIP analysis: **(E)** ChIP analysis by potential transcription factors on OXTR promoter, *n* = 4; **(F)** ChIP analysis by potential histone methylation, *n* = 4. ^∗^*P* < 0.05, vs. LG(4d) + LG(4d)/CTL group. **(G–I)** Cells were treated by either LG(4d) + LG(4d)/CTL or HG(4d) + LG(4d)/CTL, or the cells were infected on day 4 by either ERβ expression lentivirus [HG(4d) + LG(4d)/↑ERβ] or ERβ lentivirus knockdown [LG(4d) + LG(4d)/shERβ]; the cells were then harvested for biomedical analysis: **(G)** mRNA analysis, *n* = 4. **(H)** Protein quantitation, *n* = 5. **(I)** Representative western blotting pictures for **(H)**. ^∗^*P* < 0.05, vs. LG(4d) + LG(4d)/CTL group. Data were expressed as mean ± SEM.

We investigated the possible molecular mechanism for hyperglycemia-mediated OXTR suppression. A series of progressive 5′-promoter deletion constructs for the OXTR promoter were generated, and these constructs were transfected into conditional immortalized neurons for the analysis of OXTR reporter activity in the presence of either 5 mM LG or 25 mM HG for 24 h. We found that hyperglycemia-induced OXTR reporter suppression occurred among the −2000, −1600, −1400, −1200, −1100, −800, −400 and −200 deletion constructs (numbered according to Ensembl gene ID: OXTR-201 ENST00000316793.7; transcription start site was marked as 0), while suppression was significantly restored in the −1000, and −900 deletion reporter constructs, indicating that hyperglycemia-responsive transcriptional element is located in the range of −1100∼−900 on the OXTR promoter (see Figure 2A). The transcription factor database revealed many potential binding motifs, including one of the GATA1, Sp1 and YY1 and two of the C/EBPα and ERE (marked in red) binding sites located in the range of −1100∼−900 on the OXTR promoter (see Figure 2B). We then mutated these potential binding motifs in the OXTR full length (pOXTR-2000) reporter construct, and the reporter assay showed that hyperglycemia-induced reporter activation disappeared in two of the ERE mutation constructs (located at −1005 and −944, respectively, marked in green, see Figure 2B), indicating that hyperglycemia mediates OXTR suppression through the ERE binding motif on the OXTR promoter (see Figure 2C). We then made both single and double mutations on both of the ERE binding sites (located at −1005 and −944) in the pOXTR full length construct, and the reporter assay showed that ERE single mutants (M-1005/ERE, M-944/ERE) significantly decreased OXTR reporter activity in the LG treatment group compared to the wild type full length (pOXTR-2000/LG), while ERE double mutants (M-1005/-944/ERE) further decreased reporter activity, mimicking the reporter activity of the full length reporter construct (pOXTR-2000) in the HG treatment (see Figure 2D). Our results indicate that hyperglycemia induces OXTR suppression through decreased association of ERE on the OXTR promoter. We then conducted DNA methylation analysis on the OXTR promoter, and the results showed that there was no significant difference across the treatments (see Supplementary Figure 2). We then conducted ChIP analysis using antibodies for transcription factors GATA1, ERα, ERβ, C/EBPα, YY1 and Sp1 as indicated in Figure 2B. The results showed that the binding ability of ERβ on the OXTR promoter was significantly decreased in the HG(4d) + LG(4d)/CTL group compared to the LG(4d) + LG(4d)/CTL group, and this effect was completely restored by infection of SOD2 in HG(4d) + LG(4d)/↑SOD2 group; on the other hand, other transcription factors, including ERα, showed no significant difference (see Figure 2E), indicating that ERβ is responsible for hyperglycemia-induced OXTR suppression. We then evaluated the epigenetic changes in the range of −1100∼−900 on the OXTR promoter. We first evaluated the effect of hyperglycemia on histone H3 methylation. The results showed that hyperglycemia treatment had no effect on the methylation of H3K9me2 and H3K9me3, while methylation of H3K27me2 and H3K27me3 displayed a significant increase as a result of HG(4d) + LG(4d)/CTL treatment compared to the LG(4d) + LG(4d)/CTL treatment. On the other hand, infection of SOD2 in HG(4d) + LG(4d)/↑SOD2 treatment completely restored this effect (see Figure 2F). We also evaluated histone H4 methylation on the OXTR promoter (see Supplementary Figure 3a) and found that hyperglycemia did not have any effect on histone H4 methylation. We then evaluated histone acetylation on the OXTR promoter using the acetyl-histone H4 (K5, K8, K12, K16) antibody that recognizes histone H4 acetylated at lysines 5, 8, 12, or 16 and the acetyl-histone H3 (K9, K14, K18, K23, K27) antibody that recognizes histone H3 acetylated at lysines 9, 14, 18, 23 or 27 by ChIP analysis, and the results showed that there was no significant difference in either histone H3 or H4 acetylation (see Supplementary Figure 3b). We proceeded to evaluate the potential effect of ERβ on OXTR expression. The cells were infected by either ERβ expression lentivirus after HG exposure [HG(4d) + LG(4d)/↑ERβ] or ERβ knockdown lentivirus after LG exposure [LG(4d) + LG(4d)/shERβ]. The results showed that ERβ lentivirus manipulation was successful and that ERβ expression completely reversed, while ERβ knockdown mimicked hyperglycemia [HG(4d) + LG(4d)/CTL group]-induced OXTR suppression, compared to the LG(4d) + LG(4d)/CTL control group (see Figures 2G–I and Supplementary Figure 1b). Our results indicate that hyperglycemia induces OXTR suppression through epigenetic modification and the subsequent dissociation of ERβ from the OXTR promoter.

### Prenatal OXTR Deficiency Potentiates Maternal Diabetes-Mediated Oxidative Stress

**FIGURE 3:**
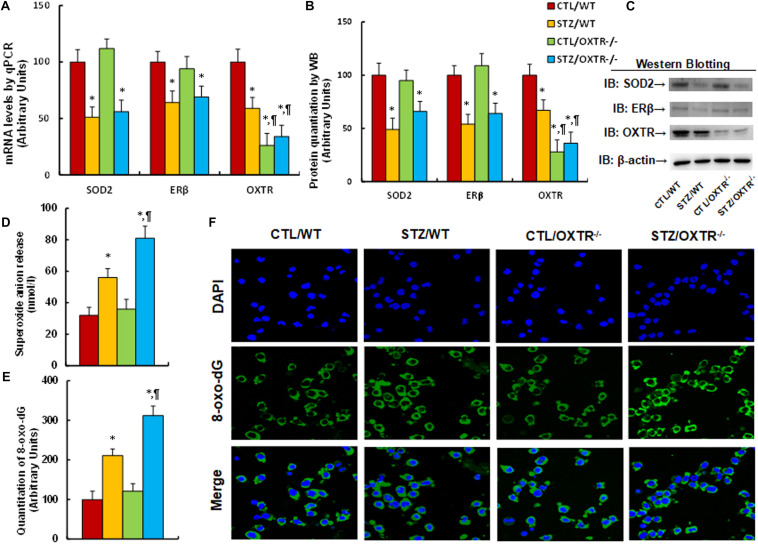
Prenatal OXTR deficiency potentiates maternal diabetes-mediated oxidative stress. The OXTR wild type (WT) or OXTR null (OXTR^–/–^) backgrounds were used to generate either control (CTL) or STZ-induced diabetic (STZ) pregnant dams, and the amygdala neurons or tissues from subsequent male offspring were isolated for further analysis. **(A–D)** The amygdala tissues were isolated from 7- to 8-week-old male offspring for analysis. **(A)** The mRNA levels by qPCR, *n* = 4. **(B)** The quantitation of protein levels, *n* = 5. **(C)** The representative pictures for western blotting for **(B)**. **(D)** *In vivo* superoxide anion release, *n* = 5. **(E,F)** The amygdala neurons were isolated on embryonic day (E18) from the above treatment for immunostaining. **(E)** Quantitation of 8-oxox-dG staining, *n* = 5. **(F)** Representative pictures for 8-oxo-dG staining (green) and DAPI staining for nuclei (blue). Two-way ANOVA was used for the statistical analysis, and each group contained nine mice. ^∗^*P* < 0.05, vs. CTL/WT group; ^¶^ *P* < 0.05, vs. STZ/WT group. Data were expressed as mean ± SEM.

We evaluated the potential effect of OXTR deficiency on maternal diabetes-mediated oxidative stress. The OXTR null (OXTR^–/–^) mice were used to generate diabetic dams through streptozocin (STZ) injection, and the brain tissues, including the amygdala, hypothalamus and hippocampus, were isolated from subsequent male offspring for further analysis. We first measured the gene expression in amygdala tissues. The results showed that gene expression of SOD2, ERβ and OXTR were significantly decreased in the maternal diabetes (STZ/WT) group compared to the control (CTL/WT) group; OXTR knockout (OXTR^–/–^) mice significantly decreased OXTR expression, but showed no effect on the expression of SOD2 and ERβ in either the control (CTL/OXTR^–/–^) or diabetic (STZ/OXTR^–/–^) groups (see Figures 3A–C and Supplementary Figure 1c). We then evaluated mRNA expression for those genes from the hypothalamus (see Supplementary Figure 4a) and hippocampus (see Supplementary Figure 4b). The results showed that the maternal diabetic (STZ/WT) group displayed significantly decreased OXTR expression levels compared to the control (CTL/WT) group, while there was no effect on the expression of SOD2 and ERβ; furthermore, OXTR expression was successfully decreased in OXTR knockout (OXTR^–/–^) mice, but there was no effect on the expression of SOD2 and ERβ. In addition, we measured OXT mRNA levels from the amygdala, hypothalamus and hippocampus, and the results showed that there was no significant difference in OXT expression across any of the treatments (see Supplementary Figure 4c). Finally, we evaluated the oxidative stress in amygdala tissues from the mice, and the results showed that maternal diabetic (STZ/WT) group displayed significantly increased superoxide anion release (see Figure 3D) and 8-oxo-dG formation (see Figures 3E,F) compared to control (CTL/WT) group; there was no effect in OXTR knockout (OXTR^–/–^) mice compared to the control (CTL/OXTR^–/–^) group, but the OXTR knockout further potentiated maternal diabetes (STZ/OXTR^–/–^) -mediated oxidative stress compared to STZ/WT group. Our results indicate that prenatal OXTR deficiency potentiates maternal diabetes-mediated oxidative stress.

### Prenatal OXTR Deficiency Potentiates Maternal Diabetes-Mediated Anxiety-Like Behavior, While It Has Little Effect on Autism-Like Behavior in Offspring

**FIGURE 4:**
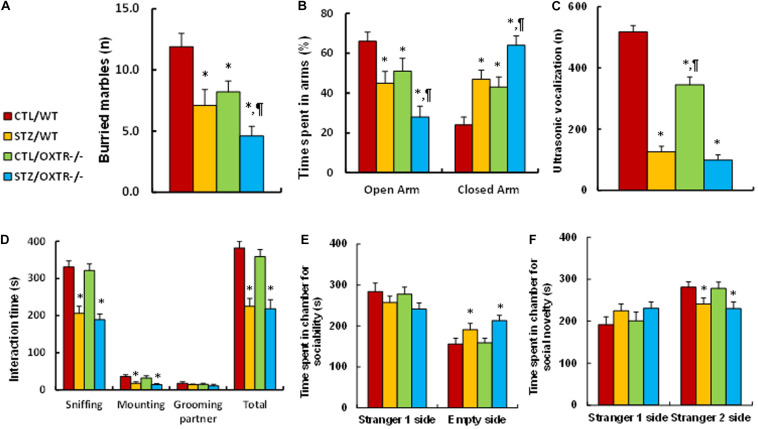
Prenatal OXTR deficiency potentiates maternal diabetes-mediated anxiety-like behavior, while it has little effect on autism-like behavior in offspring. The OXTR wild type (WT) or OXTR null (OXTR^–/–^) background were used to generate either control (CTL) or STZ-induced diabetic (STZ) pregnant dams, and the subsequent 7- to 8-week-old male offspring were used for animal behavior analysis. **(A)** Marbles burying tests (MBT), *n* = 9. **(B)** Time spent in Open Arm and Closed Arms in EPM test, *n* = 9. **(C)** Ultrasonic vocalization, *n* = 9. **(D)** Social interaction (SI) test, the time spent in following, mounting, grooming, and sniffing any body parts of the other mouse was calculated, *n* = 9. **(E,F)** Three-chambered social tests, *n* = 9. **(E)** Time spent in chamber for sociability. **(F)** Time spent in chamber for social novelty. Two-way ANOVA was used for the statistical analysis, and each group contained nine mice. ^∗^*P* < 0.05, vs. CTL/WT group; ^¶^ *P* < 0.05, vs. STZ/WT group. Data were expressed as mean ± SEM.

We evaluated the potential effect of OXTR deficiency on maternal diabetes-mediated social deficits in male offspring. We first evaluated anxiety-like behavior in these animals. The results showed that the maternal diabetic (STZ/WT) group buried significantly fewer marbles (see Figure 4A) and spent less time in the Open Arm while spending more time in the Closed Arm in EPM tests (see Figure 4B) compared to the control (CTL/WT) group. OXTR knockout mice displayed an effect mimicking that of the maternal diabetes group as compared to the control (CTL/OXTR^–/–^) group, and interestingly, it further potentiated the maternal diabetes-mediated anxiety-like behavior in diabetic (STZ/OXTR^–/–^) group compared to STZ/WT group. We then evaluated the effect of OXTR deficiency on ALBs. The results showed that maternal diabetic (STZ/WT) group had significantly fewer ultrasonic vocalizations compared to the control (CTL/WT) group. OXTR knockout mice slightly but significantly mimicked the effect of maternal diabetes in the control (CTL/OXTR^–/–^) group, while there was no further effect in the diabetic (STZ/OXTR^–/–^) group (see Figure 4C). In addition, our results showed that mice from the maternal diabetic (STZ/WT) group spent significantly less time in Sniffing, Mounting and interacting in Total, but not in Grooming their partner in the Social Interaction tests (see Figure 4D). Additionally, they spent significantly more time in the Empty side for sociability (see Figure 4E), and less time for social novelty (see Figure 4F) in three-chambered social tests, compared to the control (CTL/WT) group. However, there was no significant effect in the OXTR knockout (OXTR^–/–^) group. Our results indicate that prenatal OXTR deficiency potentiates maternal diabetes-mediated anxiety-like behavior, while it has little effect on ALB in male offspring.

### Increasing Postnatal Expression of ERβ Completely Reverses Maternal Diabetes-Induced Oxidative Stress in Offspring, While Expression of OXTR Has no Effect

**FIGURE 5:**
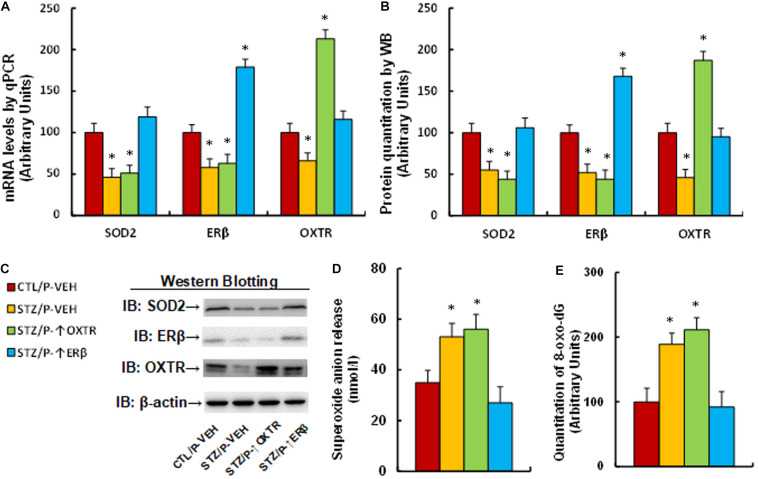
Postnatal expression of ERβ completely reverses maternal diabetes-induced oxidative stress in offspring, while expression of OXTR has no effect. The male offspring from either control (CTL) or maternal diabetes (STZ) groups received either vehicle (P-VEH), or lentivirus infusion for expression of either OXTR (P-↑OXTR) or (P-↑ERβ) at 6 weeks old, and the male offspring were sacrificed for further biomedical analysis at 8 weeks old. **(A–D)** The amygdala tissues were isolated for further analysis as below: **(A)** mRNA levels by qPCR, *n* = 4. **(B)** The quantitation of protein levels, *n* = 5. **(C)** The representative pictures for western blotting. **(D)** *In vivo* superoxide anion release, *n* = 5. **(E)** The amygdala neurons were isolated at embryonic day (E18) from the above treatment for quantitation of 8-oxox-dG staining, *n* = 5. One-way ANOVA was used for the statistical analysis, and each group contained nine mice. ^∗^*P* < 0.05, vs. CTL/P-VEH group. Data were expressed as mean ± SEM.

We evaluated the effect of postnatal expression of ERβ and OXTR on maternal diabetes-mediated oxidative stress. The male offspring from diabetic dams received expression lentivirus infusion for either ERβ or OXTR in the amygdala, and then the brain tissues, including the amygdala, hypothalamus and hippocampus, were isolated for further analysis. We first measured the gene expression in amygdala tissues. The results showed that gene expression of SOD2, ERβ and OXTR was significantly decreased in the maternal diabetes (STZ/P-VEH) group compared to the control (CTL/P-VEH) group; increasing postnatal expression of OXTR (STZ/P-↑OXTR) had no effect on SOD2 and ERβ, while increasing postnatal expression of ERβ (STZ/P-↑ERβ) completely reversed maternal diabetes-mediated gene suppression of SOD2 and OXTR (see Figures 5A–C and Supplementary Figure 1d). We then evaluated mRNA expression for these genes in both the hypothalamus and hippocampus. The results showed that OXTR expression was significantly decreased in the maternal diabetic (STZ/P-VEH) group based on analysis from both the hypothalamus (see Supplementary Figure 5a) and hippocampus (see Supplementary Figure 5b) compared to the control (CTL/P-VEH) group, while there was no significant effect on the expression of SOD2 and ERβ; additionally, postnatal infusion of either OXTR (STZ/P-↑OXTR) or ERβ (STZ/P-↑ERβ) in the amygdala had no effect on gene expression. Furthermore, we measured OXT mRNA from the amygdala, hypothalamus and hippocampus; the results showed that there was no difference on OXT expression in both the amygdala and hippocampus across all treatments, while OXT expression was significantly decreased in the maternal diabetes (STZ/P-VEH) group compared to the control (CTL/P-VEH) group in the hypothalamus, and increasing postnatal expression of either OXTR or ERβ had no effect (see Supplementary Figure 5c). Finally, we evaluated oxidative stress in the mice. The results showed that mice from the maternal diabetic (STZ/P-VEH) group had significantly increased superoxide anion release (see Figure 5D) and 8-oxo-dG formation (see Figure 5E) compared to the control (CTL/P-VEH) group, and amygdala infusion of OXTR (STZ/P-↑OXTR) had no effect, while amygdala infusion of ERβ (STZ/P-↑ERβ) completely reversed the diabetes-mediated effect. Our results indicate that increasing postnatal expression of ERβ completely reverses maternal diabetes-induced oxidative stress in offspring, while expression of OXTR has no effect.

### Increasing Postnatal Expression of OXTR Reverses Maternal Diabetes-Induced Anxiety-Like Behavior and Has Little Effect on Autism-Like Behavior, While Expression of ERβ Completely Reverses Maternal-Diabetes-Induced Social Deficits in Offspring

**FIGURE 6:**
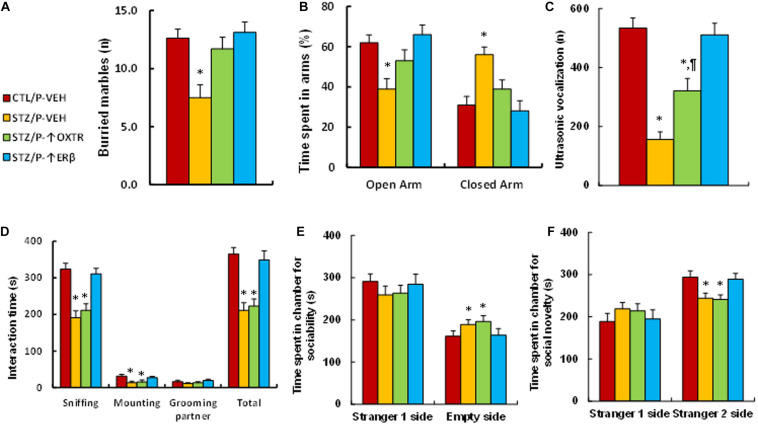
Postnatal expression of OXTR reverses maternal diabetes-induced anxiety-like behavior and has little effect on autism-like behavior, while expression of ERβ completely reverses maternal-diabetes-induced social deficits in offspring. The male offspring from either control (CTL) or maternal diabetes (STZ) groups received either vehicle (P-VEH), or lentivirus infusion for expression of either OXTR (P-↑OXTR) or (P-↑ERβ) at 6 weeks old, and the male offspring were used for animal behavior analysis at 8 weeks old. **(A)** Marbles burying tests (MBT), *n* = 9. **(B)** Time spent in Open Arm and Closed Arms in EPM test, *n* = 9. **(C)** Ultrasonic vocalization, *n* = 9. **(D)** Social interaction (SI) test, the time spent following, mounting, grooming, and sniffing any body parts of the other mouse was calculated, *n* = 9. **(E,F)** Three-chambered social tests, *n* = 9. **(E)** Time spent in chamber for sociability. **(F)** Time spent in chamber for social novelty. One-way ANOVA was used for the statistical analysis, and each group contained nine mice. ^∗^*P* < 0.05, vs. CTL/WT group; ^¶^ *P* < 0.05, vs. STZ/WT group. Data were expressed as mean ± SEM.

We evaluated the effect of postnatal expression of ERβ and OXTR on maternal diabetes-mediated social deficits in male offspring. We first evaluated anxiety-like behaviors in these animals. The results showed that mice from the maternal diabetic (STZ/P-VEH) group buried significantly fewer marbles (see Figure 6A) and spent less time in the Open Arm while spent more time in the Closed Arm in EPM tests (see Figure 6B) compared to the control (CTL/P-VEH) group; amygdala infusion of either OXTR (STZ/P-↑OXTR) or ERβ (STZ/P-↑ERβ) completely reversed the maternal diabetes-mediated effect. We then evaluated the effect of postnatal expression in the amygdala on ALB. The results showed that mice from the maternal diabetic (STZ/P-VEH) group had significantly fewer ultrasonic vocalizations compared to the control (CTL/P-VEH) group; amygdala infusion of OXTR (STZ/P-↑OXTR) partly, while amygdala infusion of ERβ (STZ/P-↑ERβ) completely, reversed the maternal diabetes-mediated effect (see Figure 6C). In addition, our results showed that maternal diabetic (STZ/P-VEH) group spent significantly less time in Sniffing, Mounting and socially interacting in Total, but not in Grooming their partner in Social Interaction tests (see Figure 6D). Furthermore, mice from this group spent significantly more time in the Empty side for sociability (see Figure 6E) and less time for social novelty (see Figure 6F) in the three-chambered social tests compared to the control (CTL/P-VEH) group; amygdala infusion of OXTR (STZ/P-↑OXTR) showed no effect, while amygdala infusion of ERβ (STZ/P-↑ERβ) completely reversed the maternal diabetes-mediated effect. Our results indicate that increasing postnatal expression of OXTR in amygdala reverses maternal diabetes-induced anxiety-like behavior but has little effect on ALB, while expression of ERβ completely reverses maternal-diabetes-induced social deficits in offspring.

## Discussion

In this study, we found that OXTR is suppressed by hyperglycemia-mediated epigenetic changes and the subsequent dissociation of ERβ from the OXTR promoter. Prenatal OXTR deficiency potentiates maternal diabetes-mediated anxiety-like behavior but has little effect on ALB; additionally, postnatal OXTR expression partly, while postnatal ERβ expression completely, reversed maternal diabetes-mediated social deficits.

### Maternal Diabetes-Mediated OXTR Suppression

We found that hyperglycemia suppresses the expression of both OXT and OXTR, and OXTR expression remains low, while OXT expression returns to normal during subsequent normoglycemia. This effect can be completely reversed by SOD2 expression, indicating that hyperglycemia-induced OXTR suppression is due to hyperglycemia-induced consistent oxidative stress, which has been termed “hyperglycemia memory” (El-Osta et al., 2008; Lu et al., 2020). Further investigation showed that hyperglycemia-induced OXTR suppression is due to oxidative stress-mediated consistent histone methylation on the OXTR promoter, indicating that these types of epigenetic changes can be inherited in offspring as a result of maternal diabetes. This conclusion has been further supported by the results from our *in vivo* study, which showed that OXTR expression was suppressed in many brain tissues, including the amygdala, hypothalamus and hippocampus, in prenatal diabetes exposure-induced offspring. In addition, we found that high glucose suppresses OXT expression, even though this cannot be inherited in offspring, indicating that diabetes may suppress OXT-mediated physiological processes, which is consistent with previous findings (Lippert et al., 2003; Gutkowska et al., 2009; Dai et al., 2018; Ding et al., 2019).

### Role of OXTR in Maternal Diabetes-Mediated Social Deficits

We found that prenatal OXTR deficiency induces many social deficits in offspring, it mimics the effects of maternal diabetes-induced anxiety-like behavior and ultrasonic vocalization (Tsuji et al., 2020), while has little effect on ALB. Very interestingly, prenatal OXTR deficiency potentiates maternal diabetes-mediated anxiety-like behavior while again having little effect on ALB, which is consistent with previous findings that OXT is associated with anxiety, but not necessarily with ALB (Yoshida et al., 2009; Puglia et al., 2015, 2018; Duque-Wilckens et al., 2020). In addition, our results showed that prenatal OXTR deficiency does not directly trigger oxidative stress in offspring, while we have previously found that maternal diabetes-induces ALB through persistent oxidative stress and SOD2 suppression (Wang et al., 2019). Taken altogether, we suggest that OXTR may contribute to ALB through other mechanisms, such as serotonergic or glutamatergic neurons, instead of triggering oxidative stress alone (Yoshida et al., 2009; Tan et al., 2019; Wang et al., 2019). On the other hand, this study has a potential limitation due to the lack of OXTR transgenic mice, and our conclusions are made using the lack of an effect of increased OXTR expression in the amygdala, however, OXTR changes were observed in several brain regions beyond the amygdala. In this case, OXTR expression in other regions of brain may also contribute to the animal behaviors, and this needs to be further investigated.

### Role of ERβ and Epigenetic Modifications on OXTR Expression

It has been reported that genetic and epigenetic change-mediated OXTR deficiency is associated with ASD (Gregory et al., 2009), and DNA methylation (Behnia et al., 2015; Maud et al., 2018; Puglia et al., 2018) on the OXTR promoter contributes to OXTR deficiency and subsequent social deficits (Puglia et al., 2015, 2018). In this study, we found that maternal diabetes-mediated OXTR suppression is due to oxidative stress-mediated histone methylation on the OXTR promoter as opposed to DNA methylation, indicating that many different factors may contribute to ASD through different mechanisms. In addition, our study has shown that hyperglycemia-induced histone methylation dissociates ERβ from the OXTR promoter and subsequently resulting in OXTR down-regulation (Kudwa et al., 2014). Additionally, we have previously reported that maternal diabetes induces suppression of both SOD2 and ERβ, subsequently contributing to ALBs (Wang et al., 2019). In this study, maternal diabetes-mediated OXTR suppression may be partly due to histone methylation and partly due to suppressed ERβ expression, supporting our previous conclusions that ERβ may play an important role in ASD development (Zou et al., 2017; Xie et al., 2018). In addition, our preliminary study showed that maternal diabetes induces significantly decreased expression of SOD2 and ERβ in brain, resulting in more severe ALBs in male offspring compared to female offspring since male offspring have relatively much lower basal ERβ expression in brain, making male offspring more susceptible to hyperglycemia-induced damage. Furthermore, the presence of high levels of estrogen in female offspring ameliorates maternal diabetes-induced ALBs by estrogen-mediated ERβ activation (Zou et al., 2017; Wang et al., 2019). In this case, the male offspring were chosen in this study to evaluate the potential effect of maternal diabetes on animal behaviors.

## Conclusion

Oxytocin receptor is suppressed by hyperglycemia-induced persistent oxidative stress and epigenetic changes, which can be inherited during subsequent normoglycemia. Maternal diabetes-induced OXTR suppression contributes to anxiety-like behavior, while it has less of an effect on ALB; moreover, prenatal OXTR deficiency potentiates maternal diabetes-mediated social deficits. We conclude that maternal diabetes-induced suppression of oxytocin receptor contributes to social deficits in offspring.

## Data Availability Statement

The original contributions presented in the study are included in the article/Supplementary Material, further inquiries can be directed to the corresponding authors.

## Ethics Statement

The animal study was reviewed and approved by the Institutional Animal Care and Use Committee from Kangning Hospital of Shenzhen.

## Author Contributions

PY wrote the manuscript. PY and JLu designed, analyzed the data, and interpreted the experiments. YL, JX, YS, LL, SS, and ZX performed vector constructions and gene expression analysis. XJ, ZW, YN, and HZ performed statistical analysis and part of the mouse experiments. JLi and YLi performed the remaining experiments. All authors read and approved the final manuscript.

## Conflict of Interest

The authors declare that the research was conducted in the absence of any commercial or financial relationships that could be construed as a potential conflict of interest.

## Footnotes

1 http://www.urogene.org/cgi-bin/methprimer/methprimer.cgi

## Abbreviations

ALB: autism-like behavior
ASD: autism spectrum disorders
ChIP: chromatin immunoprecipitation
ERE: estrogen response element
ER β: estrogen receptor β
O_2_.^–^: superoxide anions
ROS: reactive oxygen species
OXT: oxytocin
OXTR: oxytocin receptor
PVN: paraventricular nuclei
SOD2: superoxide dismutase 2
STZ: streptozocin.
