# Supplementary material for: Maternal Diabetes-Induced Suppression of Oxytocin Receptor Contributes to Social Deficits in Offspring
Source: Front Neurosci. 2021 Feb 9;15:634781. doi: 10.3389/fnins.2021.634781 (PMC7900564; doi:10.3389/fnins.2021.634781)
Supplement: Supplementary file 1 [file Table_1.DOCX]

**Maternal Diabetes-Induced Suppression of Oxytocin Receptor Contributes to Social Deficits in Offspring**

Jianbo Liu^1^, Yujie Liang^1^, Xing Jiang^2^, Jianchang Xu^1^, Yumeng Sun^1^, Zichen Wang^2^, Ling Lin^1^, Yanbin Niu^2^, Shiqi Song^1^, Huawei Zhang^2^, Zhenpeng Xue^1^, Jianping Lu^1,#^, Paul Yao^1,#^

**Supplemental Information**

Data S1. MATERIALS AND METHODS

**Reagents and materials**. Human Neural Progenitor Cells (NPC, #ACS-5003) were obtained from ATCC and were cultured in NPC medium as described previously (1). The mouse primary amygdala neurons were isolated and cultured in DMEM medium plus 10% fetal bovine serum (FBS), 10% heat-inactivated defined horse serum, 20mM D-glucose and 100 U/ml Pen/Strep (from Invitrogen). All cells were maintained in a humidified incubator with 5% CO_2_ at 37°C. In some experiments, the cells were conditionally immortalized using a hTERT lentivirus vector with an extended life span to achieve higher transfection efficiency and experimental stability (2, 3).

The antibodies for β-actin (sc-47778), C/EBPα (sc-365318), GATA1 (sc-266), SOD2 (sc-30080), Sp1 (sc-17824) and YY1 (sc-7341) were obtained from Santa Cruz Biotechnology. Antibodies for OXTR (#BS-1314R) was purchased from Fisher; OXT (#AB911) was purchased from Sigma; 8-oxo-dG (4354-MC-050) was purchased from Novus Biologicals; NeuN (#24307) was purchased from Cell Signaling. Antibodies for acetyl-histone H4 K5, K8, K12, and K16 (H4K5,8,12,16ac, #PA5-40084) were obtained from Invitrogen. Antibodies for ERα (ab3575), ERβ (ab3576), anti-histone H3 acetyl K9, K14, K18, K23, K27(H3K9,14,18,23,27ac, ab47915), H4K20me1 (ab9051), H4K20me3 (ab9053), H4R3me1 (ab17339), H3K9me2 (ab1220), H3K9me3 (ab8898), H3K27me2 (ab24684) and H3K27me3 (ab6002) were obtained from Abcam. 3-nitrotyrosine (3-NT) was measured using the 3-Nitrotyrosine ELISA Kit (ab116691 from Abcam) per manufacturers’ instructions. The mitochondrial fraction was isolated using a Pierce Mitochondria Isolation Kit (Pierce Biotechnology) per manufacturers’ instructions. Protein concentration was measured using the Coomassie Protein Assay Kit (Pierce Biotechnology). Luciferase activity assay was carried out using the Dual-Luciferase™ Assay System (Promega) and the transfection efficiency was normalized using a cotransfected renilla plasmid (4). Streptozocin (STZ, #18883-66-4) were obtained from Sigma.

**Construction of OXT/OXTR reporter plasmid.** Human genomic DNA was prepared from NPC cells. In order to construct OXT/OXTR reporter plasmids, the gene promoter (2kb upstream of the transcription start site plus first exon) was amplified from Ensembl gene ID: OXT-201 ENST00000217386.2 (for OXT) and OXTR-201 ENST00000316793.7 (for OXTR) by PCR and subcloned into the pGL3-basic vector (# E1751, Promega) using underlined restriction sites with the following primers: OXT forward: 5’-gcgc-acgcgt- ttg gat gcg ggc cac ctg gga -3’ (Mlu I) and OXT reverse: 5’- gtac- aagctt- ctt gcg cac gtc gag gtc cgg -3’ (Hind III); OXTR forward: 5’-gcgc- ggtacc - tgg aac ttt gag gat ttt ttt -3’ (Kpn I) and OXTR reverse: 5’- gtac- aagctt - ctg cac cga gtc cgc agg cga -3’ (Hind III). To map OXTR promoter activity, the related deletion promoter constructs were generated by PCR methods and subcloned into the pGL3-basic vector. All the vectors were verified by sequencing, and detailed information on these plasmids is available upon request (4).

**Generation of expression lentivirus***.* The lentivirus for human ERβ and SOD2 was prepared as described previously in our lab (1). The cDNA for mouse ERβ and OXTR was obtained from Open Biosystems and subcloned into the pLVX-Puro vector (from Clontech) using underlined restriction sites with the following primers: mouse ERβ forward primer: 5’- gtac- ctcgag- atg tcc atc tgt gcc tct tct -3’ (Xho1) and mouse ERβ reverse primer: 5’- gtac- tctaga- tca ctg tga ctg gag gtt ctg -3’ (Xba1); mouse OXTR forward primer: 5’- gtac - gaattc- atg gag ggc acg ccc gca gcc -3’ (EcoR1) and mouse OXTR reverse primer: 5’- gtac - tctaga- tca tgc cga gga tgg ttg aga -3’ (Xba1). The lentivirus for ERβ, OXTR, or empty control (CTL) was expressed through Lenti-X™ Lentiviral Expression Systems (from Clontech) per manufacturers’ instructions (1).

**Gene knockdown by shRNA lentivirus particles**. The shRNA lentivirus particles for human ERβ and SOD2 were prepared as described previously in our lab (1). The shRNA lentivirus plasmids for human SOD2 (sc-41655-SH), ERβ (sc-35325-SH) or non-target control (sc-108060) were purchased from Santa Cruz Biotechnology, and the related lentivirus for either ERβ and SOD2 or empty control (CTL) were expressed through Lenti-X™ Lentiviral Expression Systems (from Clontech) per manufacturers’ instructions. The purified and condensed lentivirus were used for in vitro gene knockdown. The knockdown efficiency was confirmed by more than 65% of mRNA reduction compared to the control group in cells using real time PCR (see Table S1).

**RT reaction and real-time quantitative PCR.** Total RNA from treated cells was extracted using the RNeasy Micro Kit (Qiagen), and the RNA was reverse transcribed using an Omniscript RT kit (Qiagen). All the primers were designed using Primer 3 Plus software with the Tm at 60°C, primer size of 21bp, and the product length in the range of 140-160bp (see Table S1). The primers were validated with an amplification efficiency in the range of 1.9-2.1, and the amplified products were confirmed with agarose gel. Real-time quantitative PCR was run on iCycler iQ (Bio-Rad) with the Quantitect SYBR green PCR kit (Qiagen). The PCR was performed by denaturing at 95°C for 8 min followed by 45 cycles of denaturation at 95°C, annealing at 60°C, and extension at 72°C for 10s, respectively. 1 µl of each cDNA was used to measure target genes. β-actin was used as the housekeeping gene for transcript normalization, and the mean values were used to calculate relative transcript levels with the ^ΔΔ^CT method per instructions from Qiagen. In brief, the amplified transcripts were quantified by the comparative threshold cycle method using β-actin as a normalizer. Fold changes in gene mRNA expression were calculated as 2^−ΔΔCT^ with CT = threshold cycle, ΔCT=CT (target gene)-CT(β-actin), and the ΔΔCT =ΔCT (experimental)-ΔCT (reference) (4, 5).

**Western blotting.** Cells were lysed in an ice-cold lysis buffer (0.137M NaCl, 2mM EDTA, 10% glycerol, 1% NP-40, 20mM Tris base, pH 8.0) with protease inhibitor cocktail (Sigma). The proteins were separated in 10% SDS-PAGE and further transferred to the PVDF membrane. The membrane was incubated with appropriate antibodies, washed and incubated with HRP-labeled secondary antibodies, and then the blots were visualized using the ECL+plus Western Blotting Detection System (Amersham). The blots were quantitated by IMAGEQUANT and final results were normalized by β-actin (4, 5).

**Luciferase reporter assay.** 1.0×10^5^ of treated cells were seeded in a 6-well plate with complete medium to grow until they reached 80% confluence. Cells were then cotransfected by 3µg of VEGF full length or deletion reporter constructs, together with 0.2µg of pRL-CMV-Luc *Renilla* plasmid (from Promega). Cells were then treated by either 5mM aspirin or empty control (CTL) for 24 hours. After treatment, the cells were harvested and the luciferase activity assays were carried out using the Dual-Luciferase^TM^ Assay System (Promega) The transfection efficiencies were normalized using a cotransfected *Renilla* plasmid, and the reporter activity was calculated according to manufacturers’ instructions (4).

**Chromatin immunoprecipitation (ChIP).** Cells were washed and crosslinked using 1% formaldehyde for 20 min and terminated by 0.1M glycine. Cell lysates were sonicated and centrifuged. 500µg of protein were pre-cleared by BSA/salmon sperm DNA with preimmune IgG and a slurry of Protein A Agarose beads. Immunoprecipitations were performed with the indicated antibodies, BSA/salmon sperm DNA and a 50% slurry of Protein A agarose beads. Input and immunoprecipitates were washed and eluted, then incubated with 0.2mg/ml Proteinase K for 2h at 42˚C, followed by 6h at 65˚C to reverse the formaldehyde crosslinking. DNA fragments were recovered through phenol/chloroform extraction and ethanol precipitation. A ~150bp fragment on the human OXTR promoter was amplified by real-time PCR (qPCR) using the primers provided in Table S1 (4, 5), and the amplified products were further confirmed by agarose gel.

**Measurement of ROS generation.** Treated cells were seeded in a 24-well plate and incubated with 10μM CM-H2DCFDA (Invitrogen) for 45 min at 37°C, and then the intracellular formation of reactive oxygen species (ROS) was measured at excitation/emission wavelengths of 485/530nm using a FLx800 microplate fluorescence reader (Bio-Tek). The data was normalized as arbitrary units (4, 6).

***In vivo* mouse experiments.** The animal protocol conformed to US NIH guidelines (Guide for the Care and Use of Laboratory Animals, No. 85-23, revised 1996), and was reviewed and approved by the Institutional Animal Care and Use Committee from Kangning Hospital of Shenzhen. All the experimental mice were either OXTR wild type (WT) or OXTR null (OXTR^-/-^) mice with a C57BL/6J mixed genetic background (a kind gift from Dr Haimou Zhang from Hubei University, China). In the generation of diabetic mice, adult (3-month-old) female mice with either WT or OXTR^-/-^ backgrounds were monitored for estrous cycles with daily vaginal smears. Only mice with at least two regular 4 to 5-day estrous cycles were included in the studies. Chronic diabetic female mice were induced by injection of 35 mg/kg streptozocin (STZ, 0.05 M sodium citrate, pH 5.5) after an 8-hour fasting period. Animals with blood glucose >250mg/dl were considered positive with the success rate of ~90%, while control (CTL) mice received only vehicle injection. In addition, maternal diabetes-mediated offspring has only ~30% survival due to diabetes-mediated all kinds of complications, in this case, each litter can only have 2-3 siblings, and they were then randomly separated into different groups for treatments, while the small litter size does not completely preclude its limitations of litter size effect (7).

Mouse Protocol 1 for prenatal treatment of diabetes or OXTR deficiency. Verified pregnant dams were randomly assigned to the following 4 groups: Group 1: CTL group mice with OXTR WT background (CTL/WT); Group 2: STZ mice with OXTR WT background (STZ/WT); Group 3: CTL group mice with OXTR null background (CTL/OXTR^-/-^); Group 4: STZ mice with OXTR null background (STZ/OXTR^-/-^). Neurons from the amygdala were isolated on embryonic day 18 (E18) as described below. The male offspring were separated from the dams on day 21 and fed with normal chow until 7-8 weeks old for behavior tests. Then, the offspring were sacrificed and various brain tissues, including the amygdala, hypothalamus and hippocampus, were isolated, flash frozen in dry ice, and then stored in a −80°C freezer for analysis of gene expression and oxidative stress.

Mouse Protocol 2 for postnatal manipulation of OXTR/ERβ expression**.** The male offspring (6 weeks old) from either the CTL or STZ group in Mouse Protocol 1 were anesthetized with a mixture of ketamine (90 mg/kg) and xylazine (2.7 mg/kg) and implanted with a guide cannula targeting the amygdala (26 gauge; Plastics One) (8). The following stereotaxic coordinates from the bregma were used for the amygdala: anteroposterior (AP)=-1.4, mediolateral (ML)=±3.5, dorsoventral (DV)=-5.1. Dorsoventral coordinates, which were based on the mouse brain atlas (9), were measured from the skull surface with the internal cannula extending 2mm beyond the end of the guide cannula. The cannula was attached to the skull with dental acrylic and jeweler’s screws and closed with an obturator (10). An osmotic minipump (Alzet model 2002; flow rate 0.5 μl/h; Cupertino, CA) connected to a 26-gauge internal cannula that extended 1 mm below the guide was implanted and used to deliver ORTR overexpression (↑OXTR), ERβ overexpression (↑ERβ), or vehicle (VEH) lentivirus. Vehicle consisting of artificial cerebrospinal fluid (aCSF; 140 mM NaCl, 3 mM KCl, 1.2 mM Na2HPO4, 1 mM MgCl2, 0.27 mM NaH2PO4, 1.2 mMCaCl2, and 7.2 mM dextrose, pH 7.4) was used for the infusion of the lentivirus. Infusion (flow rate 0.5 µl/h) begun immediately after placement of the minipump. 0.5μl of total 2×10^3^ cfu of lentivirus was infused for 1 hour. The experimental mice were separated into 4 groups, with 10 in each group. Group 1: CTL offspring with vehicle control lentivirus infusion (CTL/P-VEH); Group 2: STZ offspring with vehicle control lentivirus infusion (STZ/P-VEH); Group 3: STZ offspring with OXTR expression lentivirus infusion (STZ/P-↑OXTR); Group 4: STZ offspring with ERβ expression lentivirus infusion (STZ/P-↑ERβ). Cannula placement was verified histologically postmortem by the injection of 0.5μl of India ink (volume matching that of drug delivery in the experiments). Mice whose dye injections were not located in the amygdala were excluded from the data analysis. Two weeks after lentivirus infusion, the offspring were used for behavior tests followed by biomedical analysis, as indicated in Mouse Protocol 1 (5).

**Immunostaining**. The treated cells were transferred to cover slips, and the cells were fixed in 4% paraformaldehyde for 20 min before being incubated with 0.3% Triton X-100 in PBS for 15 min. After blocking with 5% goat serum in PBS at room temperature for 30 min, cells were incubated with anti-mouse antibody for 8-oxo-dG (# 4354-MC-050, from Novus Biologicals) for 12 h at 4°C and subsequently with secondary antibody Alexa Fluor 488. The cover slips were then mounted by antifade Mountant with DAPI (staining nuclei, in blue). Photographs were taken using a [Confocal Laser Microscope](https://www.sogou.com/link?url=DSOYnZeCC_qw-OVKG_MsR3KENashJ6PPMhOejy_Q5JJflCntg_rzjU2lo9-QKkufX5Qp7YP6841C08P_Gzn4lQD4cR4JDdkk5sef3Ee0PfoOX3hBKf-DUA..) (Leica, 20x lens) and quantitated by Image J. software.

**In vitro primary culture of amygdala neurons**. Amygdala tissues were dissected from mice on embryonic day 18 (E18 rats). Tissues were treated with 0.05% trypsin EDTA for 15 min at 37°C. Trypsin EDTA was replaced with soybean trypsin inhibitor (Sigma) for 5 min at 37°C to stop the reaction. This was then replaced with supplemented Neurobasal A (Invitrogen) followed by mechanical dissociation. Cells were then resuspended in culture media, including Neurobasal A, B27, 1×GlutaMAX and 100 U/ml Pen/Strep (from Invitrogen), and then the cells were incubated at 37°C and 5% CO2 (11). The isolated amygdala neurons were used for immunostaining (5).

**DNA methylation analysis.** We developed a real-time PCR-based method for methylation-specific PCR (MSP) analysis on the human OXTR promoter according to the previously described method with some modifications (12-14). The genomic DNA from human #ACS-5003 cells was extracted and purified before then being treated by bisulfite modification using the EpiJET Bisulfite Conversion Kit (#K1461, Fisher). The modified DNA was then amplified using methylated and unmethylated primers for MSP that were designed using the Methprimer software: (<http://www.urogene.org/cgi-bin/methprimer/methprimer.cgi>) with the below details: Methylated primer: forward 5’- ttt gag ttt att gtt aaa gtc gt -3’, reverse 5’- aaa taa taa tat tct tcc ccg aa -3’; Unmethylated primer: forward 5’- ttt gag ttt att gtt aaa gtt gt -3’; reverse 5’- aaa taa taa tat tct tcc cca aa -3’. Product size: 147bp (methylated) & 147bp (unmethylated); CpG island size: 134bp; Tm: 64.2°C. The final methylation readout was normalized by unmethylated input PCR (15).

**Animal behavior test.** The animal behavior test of offspring was carried out at 7-8 weeks of age. Anxiety-like behavior was evaluated using the marbles burying tests (MBT) and the elevated plus maze (EPM) tests (15, 16). Autism-like behavior was evaluated using ultrasonic vocalization (USV), social interaction (SI) tests and a three-chambered social test as described below (17-19).

Marbles burying test (MBT). In brief, each mouse is placed in a clean cage (35×23×19 cm^3^) filled with wood chip bedding to a depth of 5cm containing 20 colored glass marbles (1cm diameter) placed in a 5×4 arrangement. The number of marbles buried (> 50% covered by bedding material) in 30 minutes was hand-scored by the experimenter (15, 16, 20, 21).

Elevated plus maze (EPM). All behavioral tests were performed when the mice were 10 weeks old. To investigate the presence of anxiety-like behavior in male and female offspring, the EPM test, a well-established rodent model used to characterize anxiety-like behavior, was performed. The Elevated Plus Maze Package with IR Beam Detection for mouse (Cat #: MED-ELVM-1R) was obtained from Med Associates Inc. The maze is comprised of two open and two closed arms. Dual sensors at the entrance to each goal runway allow software to differentiate between runway exploration and entrance, resulting in more accurate position detection. The mice were placed in the junction area and their movements were measured for 5 min using infrared beams installed on each arm and automatically registered by the MED-PC software (Cat #: SOF-735, Med Associates) for further analysis (10, 15).

Ultrasonic vocalizations (USV). The USV of neonates were examined during a 5-min maternal-separation paradigm on postnatal day 7. USV from individually-isolated pups were recorded using an externally polarized condenser microphone with a frequency range of 30-300kHz that was attached 15-20cm above the floor of an isolation chamber. The microphone was connected to the Avisoft-UltrasoundGate recording software (Avisoft Bioacoustics, Germany) and the pup-emitted calls were recorded to WAV sound files using parameters optimized for mice. Pups were individually placed in the sound-proof chambers and calls were recorded for 300s. Data transformation on the number of USV were analyzed using a generalized linear model with a negative binomial distribution and a log-link function (17, 18).

Social interaction (SI) test. The subjects (Test and Stranger) were separately habituated to the arena for 5min before the test. During each test, the mice were placed into the apparatus over a period of 20min and the amount of time spent following, mounting, grooming, and sniffing any body parts of the other mouse was taken as an indicator of social engagement. The social interaction time was calculated and analyzed using EthoVision XT animal tracking software (Noldus, USA) (22). The animal used as the “Stranger” was used only once and was a mouse of the same gender, weight, and age that had no previous contact with the test mice (5, 20, 21, 23).

Three-chambered social test. 7-8 week-old mice were used to assess sociability and preference for social novelty. Target subjects (Stranger 1 and Stranger 2) were 7-8 week-old mice habituated to being placed inside wire cages for 3 days prior to the beginning of testing. Test mice were habituated to the testing room for at least 45 min prior to the start of behavioral tasks. For the sociability test, the test animal was introduced to the middle chamber and left to habituate for 5 min, after which an unfamiliar mouse (Stranger 1) was introduced into a wire cage in one of the side-chambers and an empty wire cage on the other side-chamber. The test animal was allowed to freely explore all 3 chambers over a 10 min session. Following this, a novel stranger mouse (Stranger 2) was introduced into the previously empty wire cage and the test animal was again left to explore for a 10 min session. Parameters scored include time spent in each chamber and number of entries into the chambers. Time spent in each chamber and track maps were calculated using automated SMART software (1, 19).

**In vivo superoxide anion (O_2_^.-^) release**. Superoxide anion release from amygdala tissues was determined by a luminol-EDTA-Fe enhanced chemiluminescence (CL) system supplemented with DMSO-TBAC (Dimethyl sulfoxide-tetrabutyl-ammonium chloride) solution for extraction of released O_2_^.-^ from tissues, as described previously. The superoxide levels were calculated from the standard curve generated by the xanthine/xanthine oxidase reaction (6).

**Statistical analysis**. The data was given as mean ± SEM and all the experiments were performed at least in quadruplicate unless indicated otherwise. The n=4 or 5 was used for the measurements of biomedical parameters, and n=9 was used for the anaslysis of animal behaviors. The one-way analysis of variance (ANOVA) followed by the Turkey−Kramer test was used to determine statistical significance of different groups, and the two-way ANOVA followed by the Bonferroni post hoc test was used to determine the differences of two factors (e.g. OXTR deficiency and maternal diabetes) using SPSS 22 software, and a *P* value of < 0.05 was considered significant.

REFERENCES

1. Wang X, Lu J, Xie W, Lu X, Liang Y, Li M, et al. Maternal diabetes induces autism-like behavior by hyperglycemia-mediated persistent oxidative stress and suppression of superoxide dismutase 2. *Proc Natl Acad Sci U S A.* 2019;116(47):23743-52.

2. Bodnar AG, Ouellette M, Frolkis M, Holt SE, Chiu CP, Morin GB, et al. Extension of life-span by introduction of telomerase into normal human cells. *Science.* 1998;279(5349):349-52.

3. Kong D, Zhan Y, Liu Z, Ding T, Li M, Yu H, et al. SIRT1-mediated ERbeta suppression in the endothelium contributes to vascular aging. *Aging Cell.* 2016;15(6):1092-102.

4. Zhang H, Li L, Li M, Huang X, Xie W, Xiang W, et al. Combination of betulinic acid and chidamide inhibits acute myeloid leukemia by suppression of the HIF1alpha pathway and generation of reactive oxygen species. *Oncotarget.* 2017;8(55):94743-58.

5. Zou Y, Lu Q, Zheng D, Chu Z, Liu Z, Chen H, et al. Prenatal levonorgestrel exposure induces autism-like behavior in offspring through ERbeta suppression in the amygdala. *Mol Autism.* 2017;8:46.

6. Yao D, Shi W, Gou Y, Zhou X, Yee Aw T, Zhou Y, et al. Fatty acid-mediated intracellular iron translocation: a synergistic mechanism of oxidative injury. *Free Radic Biol Med.* 2005;39(10):1385-98.

7. Williams DR, Carlsson R, and Burkner PC. Between-litter variation in developmental studies of hormones and behavior: Inflated false positives and diminished power. *Front Neuroendocrinol.* 2017;47:154-66.

8. Neal-Perry G, Yao D, Shu J, Sun Y, and Etgen AM. Insulin-like growth factor-I regulates LH release by modulation of kisspeptin and NMDA-mediated neurotransmission in young and middle-aged female rats. *Endocrinology.* 2014;155(5):1827-37.

9. Heldt SA, and Ressler KJ. Localized injections of midazolam into the amygdala and hippocampus induce differential changes in anxiolytic-like motor activity in mice. *Behav Pharmacol.* 2006;17(4):349-56.

10. Hu M, Richard JE, Maliqueo M, Kokosar M, Fornes R, Benrick A, et al. Maternal testosterone exposure increases anxiety-like behavior and impacts the limbic system in the offspring. *Proc Natl Acad Sci U S A.* 2015;112(46):14348-53.

11. Hay CW, Shanley L, Davidson S, Cowie P, Lear M, McGuffin P, et al. Functional effects of polymorphisms on glucocorticoid receptor modulation of human anxiogenic substance-P gene promoter activity in primary amygdala neurones. *Psychoneuroendocrinology.* 2014;47:43-55.

12. Ogino S, Kawasaki T, Brahmandam M, Cantor M, Kirkner GJ, Spiegelman D, et al. Precision and performance characteristics of bisulfite conversion and real-time PCR (MethyLight) for quantitative DNA methylation analysis. *J Mol Diagn.* 2006;8(2):209-17.

13. Eads CA, Danenberg KD, Kawakami K, Saltz LB, Blake C, Shibata D, et al. MethyLight: a high-throughput assay to measure DNA methylation. *Nucleic Acids Res.* 2000;28(8):E32.

14. Nosho K, Irahara N, Shima K, Kure S, Kirkner GJ, Schernhammer ES, et al. Comprehensive biostatistical analysis of CpG island methylator phenotype in colorectal cancer using a large population-based sample. *PLoS ONE.* 2008;3(11):e3698.

15. Zou Y, Lu Q, Zheng D, Chu Z, Liu Z, Chen H, et al. Prenatal levonorgestrel exposure induces autism-like behavior in offspring through ERβ suppression in the amygdala. *Mol Autism.* 2017;8:46.

16. Xie W, Ge X, Li L, Yao A, Wang X, Li M, et al. Resveratrol ameliorates prenatal progestin exposure-induced autism-like behavior through ERβ activation. *Mol Autism.* 2018;9:43.

17. Silverman JL, Yang M, Lord C, and Crawley JN. Behavioural phenotyping assays for mouse models of autism. *Nat Rev Neurosci.* 2010;11(7):490-502.

18. Schaafsma SM, Gagnidze K, Reyes A, Norstedt N, Mansson K, Francis K, et al. Sex-specific gene-environment interactions underlying ASD-like behaviors. *Proc Natl Acad Sci U S A.* 2017;114(6):1383-8.

19. Moy SS, Nadler JJ, Perez A, Barbaro RP, Johns JM, Magnuson TR, et al. Sociability and preference for social novelty in five inbred strains: an approach to assess autistic-like behavior in mice. *Genes Brain Behav.* 2004;3(5):287-302.

20. Bahi A. Sustained lentiviral-mediated overexpression of microRNA124a in the dentate gyrus exacerbates anxiety- and autism-like behaviors associated with neonatal isolation in rats. *Behav Brain Res.* 2016;311:298-308.

21. Bahi A. Hippocampal BDNF overexpression or microR124a silencing reduces anxiety- and autism-like behaviors in rats. *Behav Brain Res.* 2017;326:281-90.

22. Mufford JT, Paetkau MJ, Flood NJ, Regev-Shoshani G, Miller CC, and Church JS. The development of a non-invasive behavioral model of thermal heat stress in laboratory mice (Mus musculus). *J Neurosci Methods.* 2016;268:189-95.

23. Xie W, Ge X, Li L, Yao A, Wang X, Li M, et al. Resveratrol ameliorates prenatal progestin exposure-induced autism-like behavior through ERbeta activation. *Mol Autism.* 2018;9:43.

**Table S1. Sequences of primers for the real time quantitative PCR (qPCR)**

| Gene | Species | Analysis | Forward primer (5'→3') | Reverse primer (5'→3') |
| --- | --- | --- | --- | --- |
| β-actin | Human | mRNA | gatgcagaaggagatcactgc | atactcctgcttgctgatcca |
| OXT | Human | mRNA | gctgccaggaggagaactac | ctgggagaaggtggcttcc |
| OXTR | Human | mRNA | tggatctacatgctgttcacg | gatggctcaggacaaaggag |
| ERβ | Human | mRNA | atgatgatgtccctgaccaag | acatcagccccatcattaaca |
| SOD2 | Human | mRNA | gcctacgtgaacaacctgaac | tgaggtttgtccagaaaatgc |
| OXTR | Human | ChIP | agggacaggacctcagacatt | acctatcggcctcgaaaatta |
| β-actin | Mouse | mRNA | tcttgggtatggaatcctgtg | atctccttctgcatcctgtca |
| ERβ | Mouse | mRNA | atgtgctatggccaacttctg | caagcttcctcttcagggtct |
| OXTR | Mouse | mRNA | caacccatggatctacatgct | gactcaggacgaaggtggag |
| SOD2 | Mouse | mRNA | ggcctacgtgaacaatctcaa | tcaggtttgtccagaaaatgg |

FIGURE S1

**Fig S1. Representative pictures of full blots for Western Blotting.** (a) Full blots for Figure 1e. (b) Full blots for Figure 2i. (c). Full blots for Figure 3c. (d) Full blots for Figure 5c.

FIGURE S2

**Fig S2. Potential effect of hyperglycemia and SOD2 expression on the DNA methylation on the OXTR promoter.** The ACS-5003 neurons were treated by either 4-day LG plus 4-day LG (LG(4d)+LG(4d)), 4-day HG plus 4-day LG (HG(4d)+LG(4d)), or the cells were infected on day 4 by SOD2 lentivirus (HG(4d)+LG(4d)/SOD2↑), and the cells were then used for DNA methylation on the OXTR promoter, n=4. Data were expressed as mean ± SEM.

FIGURE S3

**Fig S3. Potential effect of hyperglycemia and SOD2 expression on the histone modifications on the OXTR promoter.** The ACS-5003 neurons were treated by either 4-day LG plus 4-day LG (LG(4d)+LG(4d)), 4-day HG plus 4-day LG (HG(4d)+LG(4d)), or the cells were infected on day 4 by SOD2 lentivirus (HG(4d)+LG(4d)/SOD2↑), and the cells were then used for ChIP analysis. (a) Histone H4 methylation on the OXTR promoter, n=4. (b) Histone acetylation on the OXTR promoter using H3K9,14,18,23,27ac and H4K5,8,12,16ac antibodies, n=4. Data were expressed as mean ± SEM.

FIGURE S4

**Fig S4. Potential effect of Prenatal OXTR deficiency on maternal diabetes-induced gene expression in offspring.** The OXTR wild type (WT) or OXTR null (OXTR^-/-^) background were used to generate either control (CTL) or STZ-induced diabetic (STZ) pregnant dams, and the subsequent 7-8 weeks old male offspring were sacrificed, and the tissues for amygdala, hypothalamus and hippocampus were isolated for gene analysis. (a) mRNA levels in hypothalamus, n=4. (b) mRNA levels in hippocampus, n=4. (c) OXT mRNA levels by qPCR, n=4. *, *P*<0.05, vs CTL/P-VEH group. Data were expressed as mean ± SEM.

FIGURE S5

**Fig S5. Potential effect of postnatal expression of OXTR and ERβ on maternal diabetes-induced gene expression in offspring.** The male offspring at 6 weeks old from either control (CTL) or maternal diabetes (STZ) groups received either vehicle (P-VEH), or lentivirus infusion for expression of either OXTR (P-↑OXTR) or (P-↑ERβ), and the 8-week-old male offspring were sacrificed, and the tissues for amygdala, hypothalamus and hippocampus were isolated for gene analysis. (a) mRNA levels in hypothalamus, n=4. (b) mRNA levels in hippocampus, n=4. (c) OXT mRNA levels by qPCR, n=4. *, *P*<0.05, vs CTL/P-VEH group. Data were expressed as mean ± SEM.
